# Effectiveness of a Community Empowerment Intervention to Improve Access to Pre-exposure Prophylaxis in Migrant Women Sex Workers: Protocol for a Mixed Methods Implementation Study

**DOI:** 10.2196/42844

**Published:** 2023-08-04

**Authors:** Emilie Mosnier, Fernanda Artigas, Elodie Richard, Maxime Hoyer, David Michels, Stephanie Vandentorren, Gabriel Girard, Nicolas Nagot, Hippolyte Regnault, Marine Mosnier, Grâce Inegbeze, Perrine Roux, Bruno Spire, Carole Eldin

**Affiliations:** 1 Aix Marseille University, Inserm, IRD, Sciences Economiques & Sociales de la Santé & Traitement de l’Information Médicale (SESSTIM), Aix Marseille Institute of Public Health ISSPAM Marseille France; 2 University of Health and Science, ANRS | MIE site Phnom Penh Cambodia; 3 Prospective Cooperation NGO Marseille France; 4 Université de Bordeaux; Laboratoire Bordeaux Population Health (BPH), Inserm U1219 Bordeaux France; 5 Fnasat-GV Paris France; 6 Laboratoire de recherche Communautaire, Coalition PLUS AIDES NGO Pantin France; 7 Santé publique France Saint Maurice France; 8 Pathogenesis and Control of Chronic & Emerging Infections, University of Montpellier, Inserm, Etablissement Français du Sang, University of Antilles-Guyane Montpellier France; 9 Unité des Virus Émergents (UVE) Aix-Marseille Univ-IRD 190-Inserm 1207-IHU Méditerranée Infection) Marseille France

**Keywords:** community empowerment, sexual health, implementation science, migrants, women, sex workers, mixed methods, pre-exposure prophylaxis, PrEP, treatment, intervention, France, care, healthcare, health care, community

## Abstract

**Background:**

The World Health Organization recommends pre-exposure prophylaxis (PrEP) for all populations at substantial risk of HIV infection. However, at-risk women very rarely use PrEP in France—this represents a critical issue among migrant women sex workers (MWSWs). Previous studies on PrEP use among women sex workers or migrants focused on individual or social determinants of motivation. However, operational studies in real-word settings using a holistic population approach to maximize PrEP adherence among MWSWs are lacking.

**Objective:**

FASSETS (ie, “Favoriser l’Accès à la Santé Sexuelle des Travailleuses du Sexe”; English: “facilitate the access to Sexual Health in women sex workers”) is a participative, multilevel, mixed methods study aiming to improve global knowledge of and access to sexual health care and PrEP among MWSWs through targeted empowerment strategies.

**Methods:**

This study comprises several phases: (1) phase 1: an initial qualitative study combining semistructured interviews, informal interviews, and participative observations will be performed among MWSWs, local community nongovernmental organizations, and institutions providing sexual reproductive health services to identify the determinants of PrEP access among MWSWs and for respondent-driven sampling (RDS); (2) phase 2: the size of the hidden MWSW population is estimated in Marseille through capture-recapture (the RDS survey will serve as “recapture”); (3) phase 3: a longitudinal cohort will be formed through RDS to represent the MWSW population with a goal of 150 inclusions—this cohort will be followed up for 12 months, and sequential questionnaires exploring medical history; knowledge of sexual health, HIV, and sexually transmitted infections; migration route; and current living conditions will be administered at inclusion (month 0) and months 3, 6, and 12 to measure the following interventional phase’s outcomes; and (4) phase 4: an interventional study with community empowerment actions about sexual health and PrEP will be conducted with community health workers; standardized questionnaires and semistructured interviews, observations, and focus groups will highlight MWSWs’ experiences with empowerment resources, concerns about sexual health, and especially PrEP use or uptake, and we will evaluate whether and how community-adapted empowerment actions conducted by community health workers are effective in increasing access to sexual health, prevention and screening of sexually transmitted infections, and PrEP knowledge and access among MWSWs.

**Results:**

Recruitment commenced on March 1, 2022. We estimate the follow-up period to end on September 30, 2023.

**Conclusions:**

This multiphase study will provide robust evidence about the magnitude of the MWSW population in Marseille (the second largest town in France) and their current conditions of living, access to and knowledge of sexual health, and PrEP access. Using a mixed methods analysis, we will investigate whether individual and collective community health empowerment approaches can facilitate access to PrEP and its initiation, use, and adherence in this vulnerable population.

**International Registered Report Identifier (IRRID):**

DERR1-10.2196/42844

## Introduction

In contrast to the population of men who have sex with men, where HIV incidence has decreased owing to pre-exposure prophylaxis (PrEP—taking antiretroviral drugs to prevent HIV infection), estimates of HIV incidence have not decreased in migrant populations in France and other European countries [1,2]. Indeed, the highest incidence of HIV infection has been currently reported in migrants in France [2]. Moreover, when living with HIV, migrants also tend to be diagnosed later than nonmigrants [3,4]. In this context, a recent French study has shown that a majority of migrants (58%) contracted HIV within 6 years after arrival [5], confirming previous results showing that migrants living with HIV in Europe frequently contracted the virus post migration [6-8]. In this French study, HIV acquisition after migration was linked to short or transactional partnerships and the lack of a residence permit [5]. These results highlight the economic and social determinants of HIV exposure in this population. Another study showed that in France, the majority of migrants do not know about PrEP as a tool for HIV prevention [9].

HIV prevalence among cis- or transgender women engaged in transactional sex (women sex workers [WSWs]) is substantially higher than that in similarly aged women worldwide [10,11]. Consequently, migrant WSWs (MWSWs) are at a particularly high risk of HIV infection, and progress toward the global target of HIV elimination will not be possible without interventions and programs aimed at them.

Previous studies on HIV prevention programs have specifically targeted WSWs, and PrEP has emerged as a potentially effective option for HIV prevention in this population, with most studies being performed in transitional countries [12]. PrEP is highly effective in reducing HIV incidence in this population with appropriate individual compliance [13]. Unfortunately, 2 PrEP trials among cisgender women in Africa were stopped early due to suboptimal adherence [14,15]. The subject of adherence and its determinants is of tremendous importance in this population [12]. As with HIV risk factors, determinants of PrEP use in WSWs arise from multiple social and biological factors [9,14,16]. MWSWs face structural factors associated with a precarious immigration status, migration, and occupation-related marginalization [11]. A systematic review among WSWs has stressed the need for comprehensive care in order to increase the use of and adherence to PrEP [12,17]. However, few studies have investigated how to implement community-based interventions to promote PrEP access and health equity within this vulnerable population [11]. Indeed, there is a need to develop interventions with a holistic approach, including specific clinics and social services [12,18]. Another key element is to implement a prevention intervention based on community empowerment, in which MWSWs take collective ownership of knowledge of and resources about sexual health [19].

In France, as in other European countries and the United States, women represent a minority of PrEP users (less than 3%) [20-22]. Marseille is France’s second largest city and one of the poorest (with 200,000 people living with less than €1000 [US $1086.26] per month) located on the Mediterranean coast [23]. In 2018, overall 12% of Marseille’s population (192,330 inhabitants) was born abroad and statistically considered a migrant population with 28% of them born in northern Africa and 14% of them born in sub-Saharan Africa [24]. These figures do not take into account undocumented migrants. Among 2197 people consulting for PrEP at the 10 medical centers for sexual health and prevention (managed by the departmental council of the Bouches du Rhône—an area of Marseilles and its suburbs) between 2016 and 2022, only 7 were MWSWs [25].

The objectives of the FASSETS (Favoriser l’Accès à la Santé Sexuelle des Travailleuses du Sexe) study are to (1) analyze the individual and structural determinants of the access to sexual health care and notably PrEP among MWSWs working in Marseille, (2) estimate the size of the MWSW population in Marseille, and (3) follow up with a representative cohort of 150 MWSWs to evaluate the effectiveness of a holistic approach based on community empowerment interventions targeting MWSWs to improve PrEP use.

For this purpose, we designed a mixed methods study comprising the following: (1) a study in the field with community health workers (CHWs) evaluating the determinants for the access to PrEP, (2) a capture-recapture method with respondent-driven sampling (RDS) to estimate the size and characteristics of a hidden and stigmatized population, (3) formation of a longitudinal cohort of MWSWs that will be followed up for 12 months, and (4) an intervention study involving CHWs to apply an empowerment strategy and evaluate its results with regard to the number of MWSWs taking PrEP at the end of the follow-up period.

## Methods

### Overview

**Figure 1:**
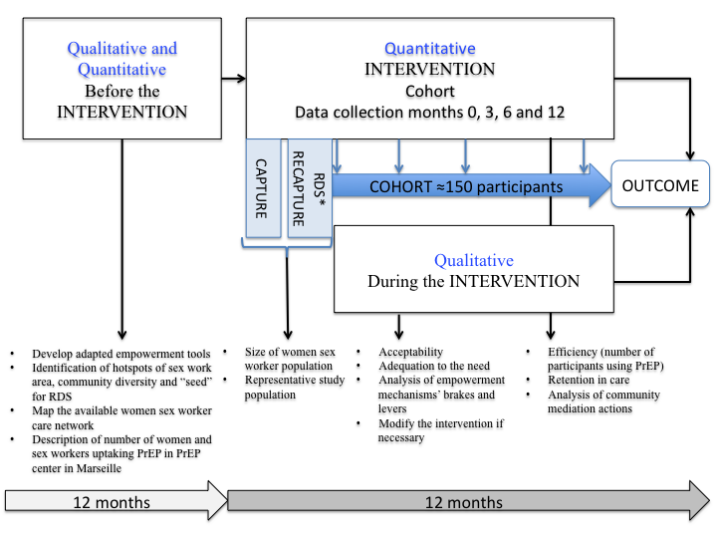
Mixed methods model of intervention analysis. PrEP: pre-exposure prophylaxis; RDS: respondent-driven sampling.

Phase 1 of the FASSETS study is a qualitative multilevel evaluation of facilitators and barriers to PrEP access for MWSWs in Marseille, which will last 12 months before the intervention begins (Figure 1).

Phase 2 will be a capture-recapture study to evaluate the size of the MWSW population in Marseille (Figure 1).

Phase 3 will involve the formation and follow-up of a representative cohort of 150 MWSWs.

Phase 4 will be the community empowerment intervention by CHWs provided to the 150 MWSWs of the cohort recruited through RDS (Figure 1).

The intervention by CHWs will consist of facilitating knowledge and PrEP uptake but also more generally to facilitate access to health care and social services as needed, using an individual and longitudinal follow-up and collective empowerment actions.

### Objectives of the Different Phases of the Study

Phase 1 will describe the determinants of PrEP uptake in MWSWs with a multilevel approach including individual, collective, and institutional analysis.

Phase 2 will determine the size of the MWSW population in Marseille.

Phase 3 will describe attitudes, knowledge, and practices of sexual and reproductive health (SRH) among MWSWs, health care pathways and mobility, testing for sexually transmitted infections, and prevalence and frequency of screening in the MWSW population at month 0 and their evolution during follow-up.

Phase 4’s primary objective is to analyze access to PrEP and retention in care through a community empowerment follow-up of MWSWs. Secondary objectives will be to document individual and collective empowerment tools for MWSWs and to describe CHW interventions for MWSWs.

### Key Outcomes for Each Phase of the Study

The key outcomes of phase 1 will be the analysis of the verbatim responses of MWSWs and institutional actors about the determinants of PrEP use, those of phase 2 will be the estimated number of MWSWs in Marseille, those of phase 3 will be the number of MWSWs included in the longitudinal study and quantitative analysis of the questionnaire data, and those of phase 4 will be the number of consultations for sexual health, access to rights, and PrEP uptake among MWSWs before and after the interventional phase.

### Study Population

The FASSETS study will take place in Marseille, which is the second largest city in France, and a significant proportion of its population has precarious living conditions [26]. The FASSETS study was developed in collaboration with the nongovernmental organization (NGO) AIDES and a Nigerian community NGO for women (The Truth), which provides HIV prevention and testing services to MWSWs.

### Phase 1: Qualitative Assessment

Qualitative data collection will be carried out separately with a trained qualitative researcher. Qualitative interviews will be conducted following semistructured interview guides in private with selected participants representative of the cohort, in focus groups, and in a multilevel assessment with doctors and social workers in the field. These interviews will be carried out to document the determinants for PrEP access in MWSWs.

### Phase 2: Estimation of the Sample Size of the Hidden Population of MWSWs by Capture-Recapture

Population size estimation is a challenge because the MWSWs are hidden and difficult to reach. Hence, capture-recapture is recommended to estimate the size of the MWSW population in Marseille [27,28]. The estimation will be based on the following equation:

*N*=(*n*1×*n*2)/*m*

where *n*1 is the number of MWSWs who received a token (capture), *n*2 is the number of MWSWs included in the RDS study (recapture), and *m* is the number of participants common to both groups (MWSWs with a token). Tokens (safety whistle: a little emergency whistle with a flashlight) will be distributed over a period of 15 days by the NGOs and institutions but also directly to the MWSW network. Indeed, distribution of safety whistles will serve as a first “capture” and the RDS survey will serve as “recapture.” When participants visit the center for RDS enrollment, they will be asked if they had received a safety whistle; if they answer yes, the research team will ask them to identify the safety whistles from a group of photographs depicting different safety whistles. If the participants correctly identify the safety whistles from the photographs, they will be included as having received a safety whistle.

### Phase 3: Representative Cohort Formation Through RDS and Follow-up

The inclusion criteria are being a cis- or transgender woman older than 18 years, self-reporting as having changed their sex for money or other resources during the past 12 months, having been born outside of France, living or working in the city of Marseille, and agreeing to participate in the study.

Numerous studies have demonstrated the effectiveness of RDS to engage adults in HIV research, particularly in stigmatized communities [29,30]. Initial RDS participants (ie, “seeds”) will be recruited for the study to achieve good representativeness in terms of the type of community or nationality, sex work (eg, indoor, outdoor, via the internet), place of residence in Marseille, and gender (cis- or transgender women). The prior qualitative study will make it possible to clearly define the characteristics of representativeness. Approximately 150 participants are expected to be enrolled in the cohort. At the beginning, participants will be offered 3 coupons. Participants who meet the eligibility criteria will be provided with a food coupon worth €10 (US $10.86) for completing each part of inclusion and follow-up (at months 3, 6, and 12), and another food coupon worth €10 (US $10.86) for each eligible and participating peer recruited. In addition, FASSETS study participants will be given public transport tickets, free condoms, and lubricating gel.

### Phase 4: FASSETS Community Empowerment Intervention and Follow-up Program

#### Empowerment Framework

There are many definitions of participant empowerment in the literature [31]. In this study, empowerment intervention will be defined as a process by which MWSWs take collective action to achieve the most effective SRH outcomes and address social and structural barriers to their health and human rights [32]. Most effective SRH effects have been defined in accordance with national and international guidelines [33,34]. The main outcomes are related to a decrease in the number of sexually transmitted infections, unintended pregnancy and abortion, sexual violence, and an increase in the access to comprehensive, good-quality information about sex and sexuality and the ability to access sexual health care. The main goal is to mainstream migrant health access to include people-centered health services. Community empowerment programs conducted in many countries to reduce HIV risk among WSWs have previously proven their effectiveness [35]. In order to evaluate empowerment, 5 specific principles will be used, including improvement, democratic participation, community knowledge, evidence-based strategies, and capacity building [36].

#### Participant and Public Involvement

CHWs and peer workers were involved in the design of the structures survey, with special attention paid to the terms and methods of communication used during participant interviews.

Participants will be involved in the intervention phase in the design of individual and collective empowerment actions. CHWs will be involved in the dissemination plans of this study by communicating in public conferences and workshops with local and nationwide actors in the field.

#### Community-Based Participatory Research

Community-based participatory research is an approach to research that involves a collective, reflective, and systematic study in which researcher and community stakeholders engage in all steps of the research process with the goal of improving practice [37]. This approach to research is recognized as particularly useful when working with populations that experience marginalization—as is the case for MWSWs—because it supports the sharing of control over individual and group health and social conditions [37,38]. The FASSETS study was initially developed around 2 community NGOs, AIDES and The Truth, which provide SRH community interventions for MWSWs. In accordance with community-based participatory research principles, this study will involve partnership building, regular exchange among partners and community NGOs, and experience sharing among the researchers, CHW staff, and the MWSW community. A community-based participatory research approach could mitigate the intersecting forms of marginalization faced by MWSWs. Indeed, a participatory approach is of particular relevance in health equity research, in which cultural and socioeconomic differences among the community, health care givers, and researchers potentially impede the identification of problems and discovery of their causes [39]. Partnering with the community will by no means eliminate this barrier during the evaluation but can enhance understanding and increase insight into the social and physical conditions as well as policy environments that are impacting the health of the community [39,40]. During the program and results dissemination, researchers, community participants, partner NGOs, and CHWs could also participate in increasing the use of social and health service usage by MWSWs and in advocacy to improve the overall living conditions of MWSWs.

### Implementation Study

The term “implementation research” describes the scientific study of the process used in the implementation of an initiative as well as the contextual factors that affect these processes [41]. This study aims to address the factors affecting the implementation of PrEP among MWSWs (such as health literacy, poverty, access to health, or traditional beliefs), the processes of implementation themselves (proposal, distribution, and follow-up of PrEP), and the outcome of the implementation under study. Indeed, the FASSETS study should be aligned with MWSWs’ needs and respond to the particularities of different communities. The study will be conducted in a real-world setting and will consider context and other factors that influence implementation. A qualitative study will help understand and document the PrEP empowerment actions in the FASSETS cohort and to describe the community mediation implementation with its positive or negative impact on MWSWs and their needs.

### Field Implementation

**Figure 2:**
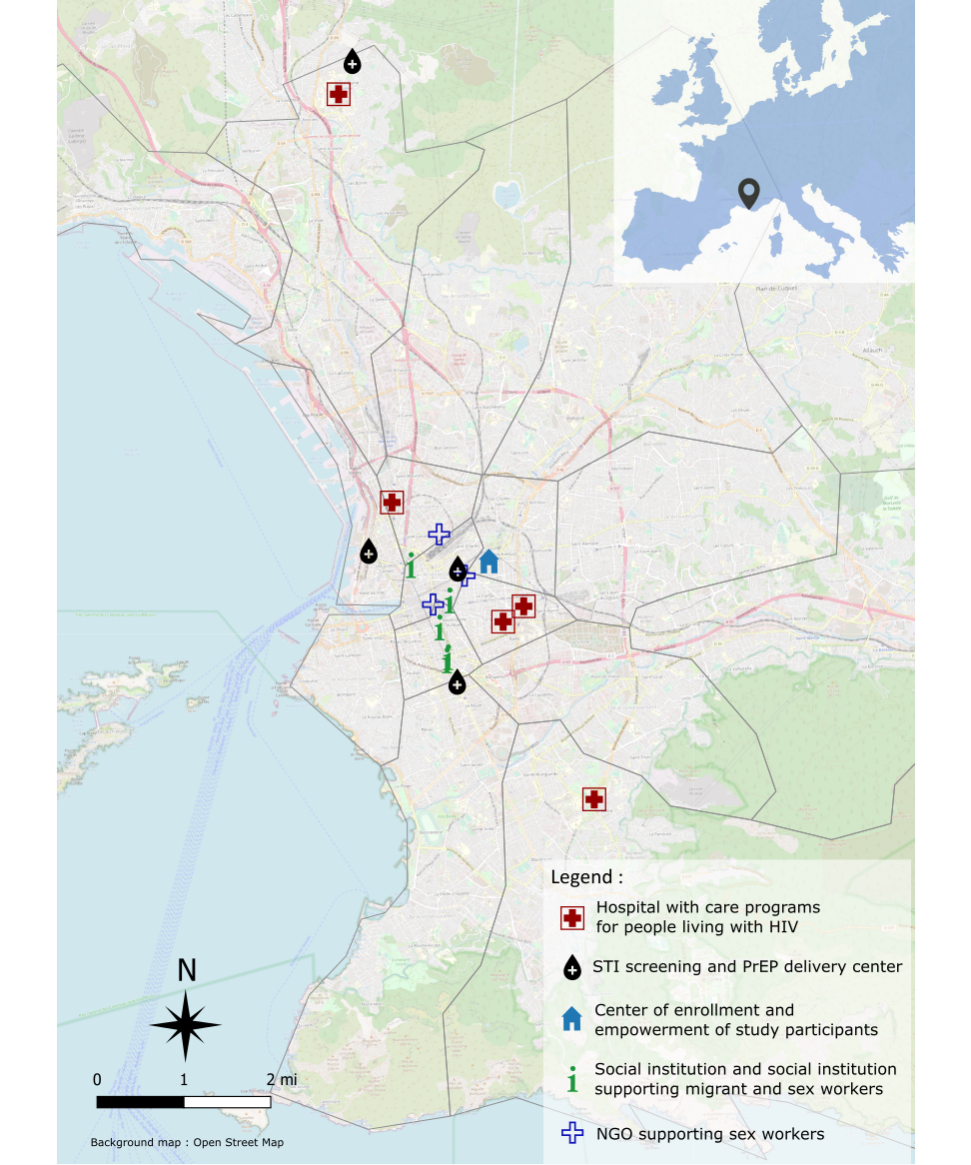
Mapping of the sexual health and social care and support network for migrant sex workers in Marseille. PrEP: pre-exposure prophylaxis; NGO: nongovernmental organization; STI: sexually transmitted infection.

The FASSETS intervention will take place at a center for enrollment and empowerment (Figure 2). The health care and social service network for MWSWs has already been mapped (Figure 2). Some NGOs and CHWs of the study are mobile and will visit some prostitution sites.

After inclusion, participants will be offered to be followed up at least every 3 months by CHWs, either in the mobile or fixed area, on the basis of their preference. Participants will also have access to health screening centers and other institutions or NGOs any time they need them, in addition to planned individual or collective meetings.

### Training of Community Health Workers and Intervention Implementers

The CHWs will be members of the communities to which the recruited MWSWs belong. They will be able to improve recruitment, accurately monitor the correct understanding of the questionnaires, and conduct and ensure participation in empowerment, prevention, and screening interventions. They will provide information about PrEP and assist with care and follow-up for participants who wish to receive the treatment. They will be uniformly trained to conduct the survey using a structured interview. All actions (type, date, and public) taken by the CHWs with MWSW participants (eg, access to care, prevention, and assistance in accessing social insurance) will be also collected.

The CHWs will be responsible for health education and individual and collective empowerment actions. They will receive rigorous prejob training and assessment, which include PrEP use and sexual health prevention and care, by caregivers (infectious disease physicians) and nationally recognized community-based NGOs working in the field of HIV prevention (including AIDES, Planning familial, and COREVIH [COordination REgionale de lute contre le Virus de l’Immunodéficience Humaine]). The training program will be conducted with the entire CHW group and will also include individualized follow-up. All CHWs will have a discussion platform via a social network to obtain real-time responses from physicians. Information or prevention tools, adapted to the MWSW populations, will be made available to the CHWs by the NGO’s partners. Posttraining mentorship, supervision, and monitoring with regular training will take place every 3 months by the investigating physicians. CHWs’ course evaluation feedback and posttraining monitoring visits will be provided by the study coordinator. Finally, a follow-up and psychological support will be set up to support the CHWs in their work with this vulnerable population.

### Data Collection

Upon enrollment and at months 3, 6, and 12, participants will be asked to complete a structured survey administered by a CHW. The survey will include questions regarding network size for RDS weighting. These will include questions about the token given at the time of data capture. Individual data including sociodemographic characteristics; sexual practices; knowledge and behavior; HIV testing history; medical history; PrEP knowledge, adherence, and compliance; contraception; drug and alcohol use; mental health (including Perceived Stress Scale-10 scores); quality of life; literacy; social support; being subjected to racism; stigmatization or violence during the past 12 months; and mobility will be collected. Other more structural determinants will be collected as follows: residence permits, health insurance, housing conditions, and food security (Table 1). Indeed, such structural determinants are previously described to have a significant impact on health care access among sex workers [42]. The semistructured questionnaire will explore the barriers and levers to accessing PrEP among MWSWs. The instrument or interview guide will attempt to understand how PrEP fits into women’s needs, care pathways, and mobility. Representation of MWSWs’ PrEP uptake among caregivers and which mechanisms are (or are not) implemented by social institutions to facilitate access to prevention and care for MWSWs (Table 1).

Type, objective, and number of actions of mediation in terms of prevention, support, or access to health care or social services will be self-reported by CHWs.

CHWs will use encrypted and secure survey software to report the MWSWs’ responses to the questionnaires and to monitor their actions of mediation. All electronic questionnaires will be forwarded to a database that is managed and hosted by SESSTIM (Inserm laboratory in Marseille). Each staff member will have a secure individual account.

Databases of all NGOs and institutional screening and PrEP centers of Marseille will be retrospectively analyzed to describe the HIV status and to assess PrEP characteristics among migrants and women in Marseille.

**Table 1:** Data collection tools and timing.

Method	Timing	Population	Data collected
Questionnaire	4 rounds: months 0, 3, 6, and 12	Randomized migrant woman sex worker cohort in Marseille	Sociodemographic data, knowledge of sexual health, results of tests for sexually transmitted diseases, medical treatment including pre-exposure prophylaxis (PrEP), retention time in care, health care pathway, residence permit, health insurance coverage, housing conditions, and food security
Questionnaire	Continuous	Community health workers	Type, number, and objective of actions
Semistructured interviews and focus groups	Continuous from September 2021 through January 2022	Migrant women sex workers	Perception of PrEP and sexual health, qualitative explanation of possible empowerment mechanisms, health needs, and care pathway
Semistructured interviews	Continuous from September 2021 through January 2022	Nongovernmental organizations (NGOs) and institutions in charge of the sexual health of sex workers in Marseille	Perception of PrEP and sexual health in migrant women sex workers, identification of available empowerment resources for migrant women, and health care offer
Observation	Continuous	NGOs and institutions in charge of the sexual health of sex workers and migrants in local and international territories	Social history of public sexual health strategies among migrant women sex workers

### Data Analysis

Descriptive statistics will be applied to the characteristics of the survey population. Categorical variables will be reported as percentages and compared using the chi-square test (and the Fisher exact test for small numbers). Continuous variables will be expressed as mean (SD) or median (IQR) values, and compared using Student and Wilcoxon signed ranked tests in accordance with the variable distribution. A simple logistic regression analysis will be applied to select variables associated with the outcome, and all variables with a *P* value of *<*.10 will be entered into a multiple logistic regression model for each outcome. Statistical analysis will be performed using STATA (version 11.2; StataCorp), and statistical significance will be defined as a *P* value of ≤.05.

Survival analysis will be used to analyze cumulative retention in care for 12 months after the beginning of the intervention. Differences between survival curves will be assessed at month 12 using the Kaplan-Meier estimate (log-rank test).

### Ethics Approval and Consent to Participate

The protocol received approval from French Ethics Committee in October 2021 (2021—A01746-35). Prior informed consent of the participants will be collected by a member of the research team before data collection. All the participants are free to withdraw from the study at any moment without incurring any penalties or consequences with regard to future care or services they might rightfully expect.

The study protocol has also been reviewed and approved by an independent peer review for funding by Sidaction.

Planned dissemination of study findings includes manuscript publication, conference presentations, and community dissemination (eg, community meetings and social network).

## Results

Recruitment commenced on March 1, 2022. We estimate that the follow-up period will end on September 30, 2023.

## Discussion

### Anticipated Findings

Community empowerment interventions that aim to reduce the risk of HIV acquisition using PrEP among MWSWs are scarce worldwide. To our knowledge, this is the first intervention of its kind in Europe. Innovative and community-based approaches to bring MWSWs closer to health and social services are required. The findings from the FASSETS study will prove useful for tailoring PrEP use, proposing PrEP use to MWSWs and ensuring its adherence among them, and developing a sustainable community-based intervention.

One of the main challenges of this study is to take into account all the barriers to access to care in this vulnerable population and possibly the low prioritization of PrEP in light of their vital needs. Indeed, MWSWs have to deal with many structural factors such as the criminalization of their work, stigmatization, racism, or their precariousness [17,42]. This project, through a holistic approach based on the needs of the participants, aims to work on the issue of empowerment in a transversal approach. CHWs will be trained in sexual health prevention and will help the participants better access their rights and basic needs, especially those of housing, civic rights (eg, resident permit and social insurance), and access to food. Regarding the fight against stigmatization and racism, this study aims to set up a collective empowerment program with the help of CHWs for better consideration of their difficulties. Partner NGOs such as The Truth and AIDES will participate in advocacy in accordance with the difficulties encountered by the participants. Moreover, CHWs’ actions have been previously described as having a social transformation impact [43].

The FASSETS intervention team has the capacity to provide cohort follow-up and individual and collective empowerment actions through partnerships with local organizations interested in connecting with the population of MWSWs. This team includes CHWs from multiple countries and cultures. FASSETS participants will have greater access to services, and health organizations may be able to reach a wider segment of the population to propose the use of PrEP.

FASSETS is a comprehensive, interdisciplinary, participatory study that combines clinical, epidemiological, and social sciences. Research questions are not limited to the participants and they take the health care network and implementing staff into consideration, notably with health mediation descriptions.

### Limitations

Operational challenges are numerous and are linked to the relationship with MWSW communities and their perception of the intervention, mobility of the participants, stigmatization and racism that they could face from institutions, decrease in preventive actions, and modification of the type of sex work due to health measures or restrictions linked to the COVID-19 pandemic. Documenting all these logistic and operational aspects should enable gathering of crucial information regarding the potential transferability of the intervention.

Regarding the FASSETS methodology of evaluation, we were unable to use a control group or a stepped wedge method because of ethical problems (benefit to participate) and the difficulty to implement empowerment in a sequential and reproducible way. However, exhaustive local data from all screening and delivery PrEP centers in Marseille are available for investigators at the beginning and at the end of the study and will permit them to evaluate, through pre- and postintervention comparisons and those between participants and nonparticipants, the access and adherence to PrEP among MWSWs.

### Conclusions

The FASSETS intervention aims to understand whether the implementation of a specific community accompaniment for MWSWs regarding PrEP use with an adapted follow-up and wide access to health care and social services are sufficient to improve the uptake of PrEP in this vulnerable population.

## Abbreviations

CHW: community health worker
FASSETS: Favoriser l’Accès à la Santé Sexuelle des Travailleuses du Sexe
MWSW: migrant women sex worker
NGO: nongovernmental organization
PrEP: pre-exposure prophylaxis
RDS: respondent-driven sampling
SRH: sexual and reproductive health
WSW: woman sex worker
